# EMAAS: An extensible grid-based Rich Internet Application for microarray data analysis and management

**DOI:** 10.1186/1471-2105-9-493

**Published:** 2008-11-25

**Authors:** G Barton, J Abbott, N Chiba, DW Huang, Y Huang, M Krznaric, J Mack-Smith, A Saleem, BT Sherman, B Tiwari, C Tomlinson, T Aitman, J Darlington, L Game, MJE Sternberg, SA Butcher

**Affiliations:** 1 Centre for Bioinformatics, Division of Molecular Biosciences, Faculty of Natural Sciences, Biochemistry Building, South Kensington Campus, Imperial College, London, SW7 2AZ, UK; 2 London e-science Centre, Department of Computing, Faculty of Engineering, William Penney Building, South Kensington Campus, Imperial College, London, SW7 2AZ, UK; 3 MRC Clinical Sciences Centre, Hammersmith Hospital Campus Imperial College London, Du Cane Road, London, W12 0NN, UK; 4 Laboratory of Immunopathogenesis and Bioinformatics, Clinical Services Program, SAIC-Frederick Inc., National Cancer Institute at Frederick, Frederick, MD 21702, USA

## Abstract

**Background:**

Microarray experimentation requires the application of complex analysis methods as well as the use of non-trivial computer technologies to manage the resultant large data sets. This, together with the proliferation of tools and techniques for microarray data analysis, makes it very challenging for a laboratory scientist to keep up-to-date with the latest developments in this field. Our aim was to develop a distributed e-support system for microarray data analysis and management.

**Results:**

EMAAS (Extensible MicroArray Analysis System) is a multi-user rich internet application (RIA) providing simple, robust access to up-to-date resources for microarray data storage and analysis, combined with integrated tools to optimise real time user support and training. The system leverages the power of distributed computing to perform microarray analyses, and provides seamless access to resources located at various remote facilities. The EMAAS framework allows users to import microarray data from several sources to an underlying database, to pre-process, quality assess and analyse the data, to perform functional analyses, and to track data analysis steps, all through a single easy to use web portal. This interface offers distance support to users both in the form of video tutorials and via live screen feeds using the web conferencing tool EVO. A number of analysis packages, including R-Bioconductor and Affymetrix Power Tools have been integrated on the server side and are available programmatically through the Postgres-PLR library or on grid compute clusters. Integrated distributed resources include the functional annotation tool DAVID, GeneCards and the microarray data repositories GEO, CELSIUS and MiMiR. EMAAS currently supports analysis of Affymetrix 3' and Exon expression arrays, and the system is extensible to cater for other microarray and transcriptomic platforms.

**Conclusion:**

EMAAS enables users to track and perform microarray data management and analysis tasks through a single easy-to-use web application. The system architecture is flexible and scalable to allow new array types, analysis algorithms and tools to be added with relative ease and to cope with large increases in data volume.

## Background

The post-genomic technologies, such as transcriptomics and proteomics, are continuing to produce new challenges for the biological community. All new technologies require new skills for interpretation, but in addition the 'omic technologies require expertise in data-management and exploration of multivariate data. All too often researchers are unable to extract the full benefit from their investment in research due to difficulties in applying best bioinformatics practice to their experiments. This problem is set to increase as more researchers adopt these high throughput methodologies, particularly with the move towards integrative and systems biology, where datasets are becoming larger, requiring extremely careful quality control at all steps, before cross-experiment data mining is meaningful or indeed possible.

Additional issues hampering the bench scientist are those of software usability and implementation in a fast-developing research area. Array technologies are under continual development e.g. exon arrays, ChIP-on-chip, SNP chip as well as data now coming online from the non-array high through-put technologies such as ChIP-Seq and more recently high throughput transcriptomic sequencing (RNA-Seq).

This is besides the wealth of statistical methods development leading to a multiplicity of new algorithms and tools, often fast-tracked to the public domain with rudimentary user interfaces or available only as scripts for command-line statistical packages such as R [1]. This leads to a time-lag in adoption for many scientists who may not have the necessary expertise or time to learn how to install or maintain multiple programs, often requiring multiple additional libraries, or to run a number of individual tools from the command-line or via custom scripts. The problem becomes more acute when we consider that the compute requirements for some analyses are becoming too large for comfortable analysis on individual desktop machines, due to a combination of dataset size and algorithm choice. For instance, the requirement to support normalisation across a hundred Affymetrix exon arrays, each with up to 5 million data points using for example the RMA pre-processing algorithm, is by no means unusual in a microarray experiment, but this could be too demanding for the hardware resources available on a desktop computer. This situation can be addressed by harnessing distributed computing resources, but again the start-up time investment is beyond the scope of most individual end-users.

To facilitate microarray data analysis and management we have developed EMAAS (Extensible MicroArray Analysis System), a multi-user Rich Internet Application (RIA), utilising a distributed computing back-end. The EMAAS infrastructure supports:

◦ Data transfer between specialised sites and data repositories

◦ Single point access using a bespoke portal to a range of tools and packages for microarray analysis with seamless data flow between the various tools

◦ Fast-track easy access for biological researchers via the portal to new models and algorithms developed in-house and externally e.g. by statisticians, computer scientists via modular wrapper implementations

◦ Automated detailed tracking of all the analysis steps performed

◦ Storage of raw data, analysis steps and analysed data in an underlying relational database

◦ Ability to access online live expertise in data analysis from local support services for researchers, including remote audio-visual interaction between researchers and staff on different sites (e.g. shared live screen views, audio-video). This can be extended to include access for collaborating experts in other specialities e.g. statisticians.

The system builds upon several open-source technologies already available for microarray data analysis, combining them to form a fully integrated user-friendly system. This allows the user to perform data management and analysis tasks through a single web interface.

Numerous microarray tools are already available for various stages of the microarray data analysis workflow, including several client-server based tools such as the commercial packages GeneSpring GX Workgroup[2](licensed by Agilent) and Resolver[3] (licensed by Rosetta) and freely available packages such as ExpressionProfiler[4], GenePattern[5] and Gecko[6]. Each has its own advantages and disadvantages with respect to parameters such as cost, ability to handle concurrent users in a multi-user environment, scalability, and ease of use of the user interface.

The aim of this project was not to re-write these tools in a static closed system, but to build a modular flexible framework that allows single-point access to existing tools and specialist websites running both on the local server and remotely, and to enable new algorithms, methods and web services to be added as and when they are developed. This enables a user to perform their analysis from start to finish through a single user interface, using the most appropriate data handling and analysis tools, without the requirement to continually update and install multiple programs on their own desktop machine. The system was designed specifically to support microarray analyses and was optimised with this in mind.

## Implementation

### Overview

EMAAS is a web based tool developed using one of the latest internet technologies, resulting in a user-friendly and interactive interface accessible from any of the most popular web browsers. All the data analysis takes place on the server side and the user does not have to install any software on their personal computer.

The interface is designed to guide the user through a standard microarray analysis workflow. Each stage of the analysis is tracked, with the results and algorithms, and parameters saved to a database on the server, allowing users to re-visit their data analysis results at a later date and enabling support staff to view the analysis workflow. The interface also includes access to interactive video tutorials and a remote support tool, which allows users and support staff to communicate with each other and to view each others user interface.

On the server side, EMAAS can interact with 3^rd ^party analysis resources located locally, such as R and the Affymetrix Power Tools, or resources hosted further afield at remote facilities such as public data repositories, through the use of distributed computing services. This architecture allows multiple users to access EMAAS resources at the same time as well as enabling users and support staff to share data. It also allows the functionality of the system to be extended with minimal disruption to the users.

The implementation of various technologies integrated into EMAAS will be described in more detail in the following sections. See table 1 for a list of resources and tools integrated into EMAAS.

**Table 1:** List of Resources Integrated into EMAAS

**Resource Type**	**Resource**	**Further Details**	**Version**
Client side web technology	OpenLaszlo		3.3.4

Web server	Apache-Tomcat		6.0.13
	Java & JSP's/Servletts		1.5
	xvfb-run		

Database server	Postgres		8.2.4
	PLR		0.6

Analysis tools (Installed on local resources)	R		2.5.1
	Bioconductor		2
	Affymetrix Power Tools		1.8
	Exonmap		1.2.02

Distributed Resources	MiMiR		-
	CELSIUS		-
	GEO		-
	EVO		-
	DAVID		-
	GeneCards		-

Grid Resources	GridSAM		-
	GridEngine		-

Development Tools	Eclipse		-
	SVN		-
	Trac		

### EMAAS Architecture

#### User Interface

**Figure 1:**
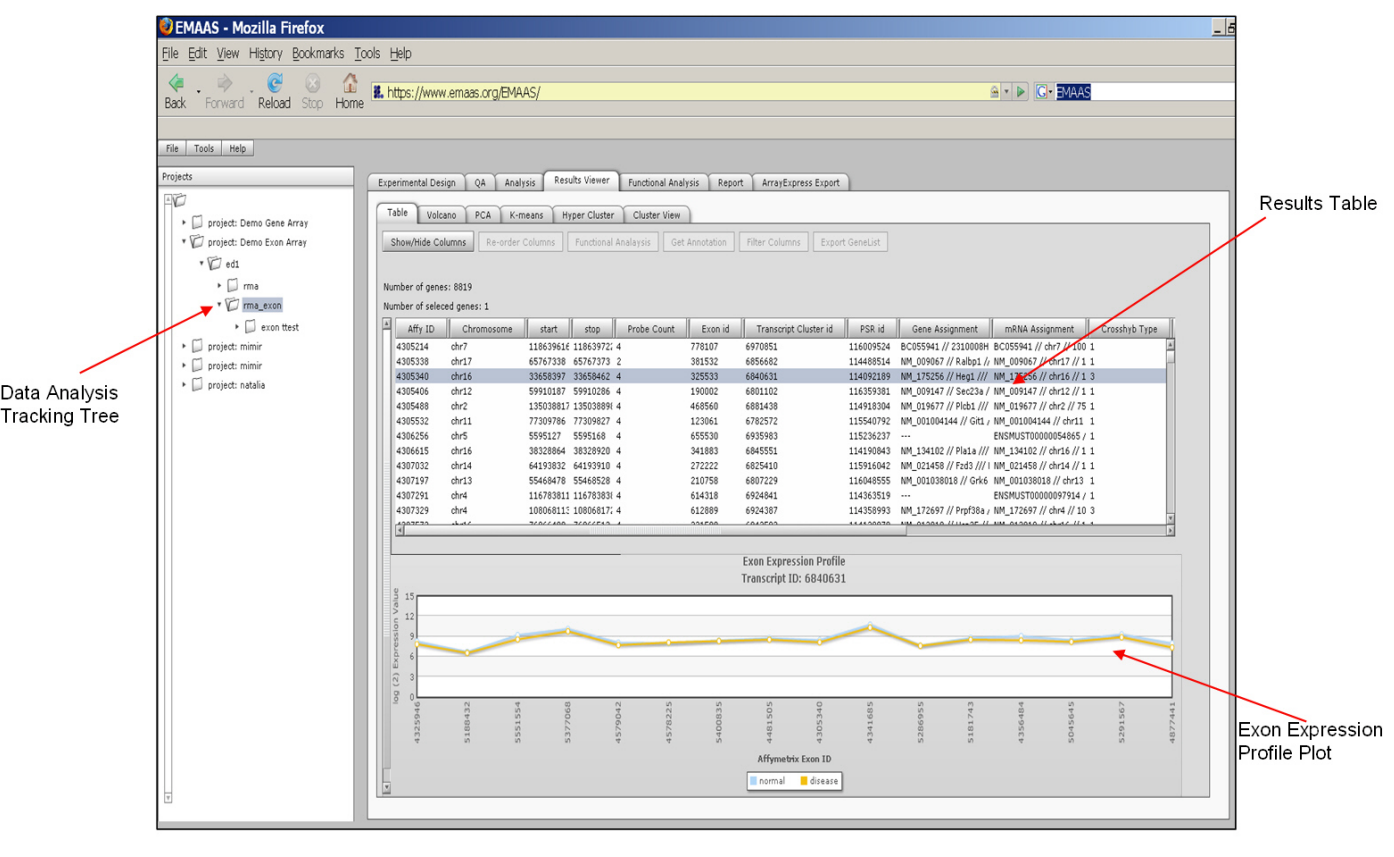
Screen shot of the EMAAS user interface showing the analysis results view and analysis tracking tree.

The user interface (as shown in figure 1) was developed using the web-based Rich Internet Application (RIA) framework OpenLaszlo [7]. OpenLaszlo is an open source platform for creating zero-install web applications, which results in the end user not having to deal with any complex installation issues.

Laszlo code is compiled into a Flash executable and this gives the interface cross platform compatibility, as such, EMAAS can be used in any browser that has Flash capabilities, which according to a recent survey is over 98% of Internet-enabled computers [8]. This includes access through some of the most commonly used browsers such as Internet Explorer, Firefox and Safari, running on Windows, Mac and Linux operating systems. Although the EMAAS interface is web-based, Laszlo provides many of the capabilities and the look and feel of standalone desktop software, allowing a feature-rich, user friendly interface to be developed, with interactive control and visualisations.

Considerable research into the preferences and work practices of researchers with varied levels of expertise in microarray analysis was used to inform the design of the user interface. We believe that this has resulted in a system that will be equally suited to those just starting to learn microarray analysis, or occasional users, as well as those with more experience, or performing larger, more complex analyses. The interface also enables users to send feedback and comments to EMAAS administrators in real-time, either through emails to a help address, or through a suggestion box integrated into the EMAAS interface.

#### Administration Interface

EMAAS is a multi-user application. An administrative interface is available for users who have EMAAS administration rights and is used to set data access privileges for users at the individual or group level. This enables users who are working together on the same project to share their data and the analysis results if required. It also allows 'privileged' support staff to gain access to user's data and analysis pipelines in order to see how the analysis has been performed and to carry out alternative methods of analysis on the same data set where appropriate.

#### Server Side

The EMAAS web server is based on Apache-Tomcat. This contains the Laszlo compilation and runtime engine. The EMAAS-Laszlo user interface interacts with the server side components, such as the EMAAS database, grid and distributed resources though Java Server Pages and Java servlets. Data and additional information are fed back to the Laszlo interface as dynamically generated XML.

**Figure 2:**
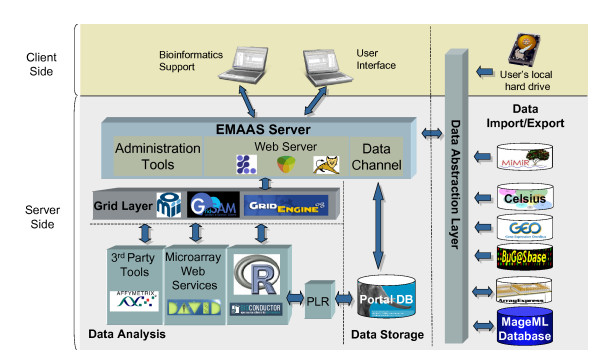
Schema of the EMAAS architecture.

Several data analysis software packages have been integrated into EMAAS, see Figure 2 for an overview of the system architecture. The main analysis tool integrated to date is R together with Bioconductor [9]. Several technologies for programmatically calling R were assessed including Rserve [10], Taverna [11], Java system calls and the Postgres PLR library [12].

For automated data analysis, the Postgres PLR library has been implemented. PLR allows R functions to be called programmatically within SQL statements. One of the main reasons for using PLR is that it allows data to be passed directly from the database to the R environment and back again in a robust manner. Thus the results from each step of the data analysis, in the form of an R Data Frame for example, can be easily serialised into the EMAAS database, which greatly facilitates data handling, data persistence and analysis tracking. Postgres is used as the underlying EMAAS database technology. To add new analysis R scripts in the current system, the R code is wrapped into a PLR function and the method details are referenced in the 'functions' database table. The Analysis user interface has then to be updated to capture the method parameters required for the new algorithm.

Also integrated in the current version of EMAAS are the Affymetrix Power Tools (APTs) [13], a set of cross-platform command-line programs that implement algorithms for analyzing and working with Affymetrix GeneChip arrays. The APTs were added in particular for processing Affymetrix Exon arrays, but can also be used for processing Affymetrix 3' GeneChips, SNP chips and Gene Expression arrays. APTs have been developed in C++ and have been designed to make efficient use of available hardware resources.

#### Distributed Computing

The system has been designed to make use of available distributed computing resources in order to speed up various steps of the data analysis workflow and to allow access to high throughput compute resources to perform analysis that may not be possible on a single desktop machine.

Data workflow tasks such as pre-processing, quality assessment and analysis require a large amount of compute power, particularly in a multi-user environment involving large data sets. Furthermore, some tasks, such as the generation of QA plots, can be done in parallel using distributed computing on grid clusters, in which case the overall duration of the plot generation and data processing can be significantly reduced.

The APTs and R-Bioinconductor have been installed throughout nodes on a compute cluster managed by a Distributed Resource Manager (DRM). In this case GridEngine [14] is used, but other DRMs, such or Condor [15], could also be integrated using the same architecture. The cluster is accessed from the web server using GridSAM [16], an open-source job submission and monitoring web service, designed and implemented in the London e-Science Centre and commissioned by the UK Open Middleware Infrastructure Institute (OMII) [17]. GridSAM endorses the WS-I set of web service standards and the Job Submission Description Language and provides a Web Service for submitting and monitoring jobs managed by a variety of DRMs. The modular design allows third-party to provide submission and file-transfer plug-ins to GridSAM.

The scalability of the system is essential, as the size of data sets increases, more users analyse their data using EMAAS and analysis methods become more sophisticated and resource hungry. This architecture allows any number of additional compute nodes to be added to provide additional compute resources for analysis steps, and together, these DRM tools transparently and seamlessly manage all job scheduling tasks without any user intervention.

#### Extensibility to other Microarray Platforms

The statistical analysis component of the pipeline takes place in an R environment and is based around the 'ExpressionSet' R class. The ExpressionSet object holds both the microarray data and the experimental meta-data and was designed to be generic enough to accommodate data from any microarray platform. A bespoke JSP function and PLR wrapper would have to be developed in EMAAS for each microarray platform, such as Illumina or Agilent, in order to import data and to create an ExpressionSet object. Then the analysis can be performed as described in the 'Data Analysis Workflow' section below.

#### Distance Support Tools

Several tools have been assessed for integration within EMAAS to provide real time analysis support for users. These tools include EVO[18], NetMeeting[19], VNC[20], VRVS[21], Webex[22] and Copilot[23]. Each has its own advantages (e.g. free, cross-platform compatibility) and disadvantages (cost, single platform, hard for local user to set up, and firewall issues) over the others.

The web conferencing tool EVO (Enabling Virtual Organizations) was selected as the integrated distance support tool of choice. This is a freely available open source tool that allows bioinformatics support staff to set up private meetings with a user or a number of users simultaneously. Tools are available in EVO for instant message, audio and video communication, real-time sharing of screens between user and support staff, whiteboard facility to share on the fly notes and file transfer.

As part of our ongoing commitment to usability and training, a number of short training videos have been developed and are available from the EMAAS web interface to guide users through different aspects of data analysis. Also being currently assessed is the use of several flash-based multi-media tools available in Laszlo for real time user interactivity, such as web camera API's, coupled with the open source streaming media server red5[24] to provide live screen grabs of the support staff and user's interfaces.

### Data Analysis Workflow in EMAAS

#### Data Import and Management

Data import is supported from a number of different sources. Users can upload their data from their local file system. A file system browser launched from the EMAAS interface allows a user to search and select data files on the local file system. Selected files are transferred to the EMAAS database.

**Figure 3:**
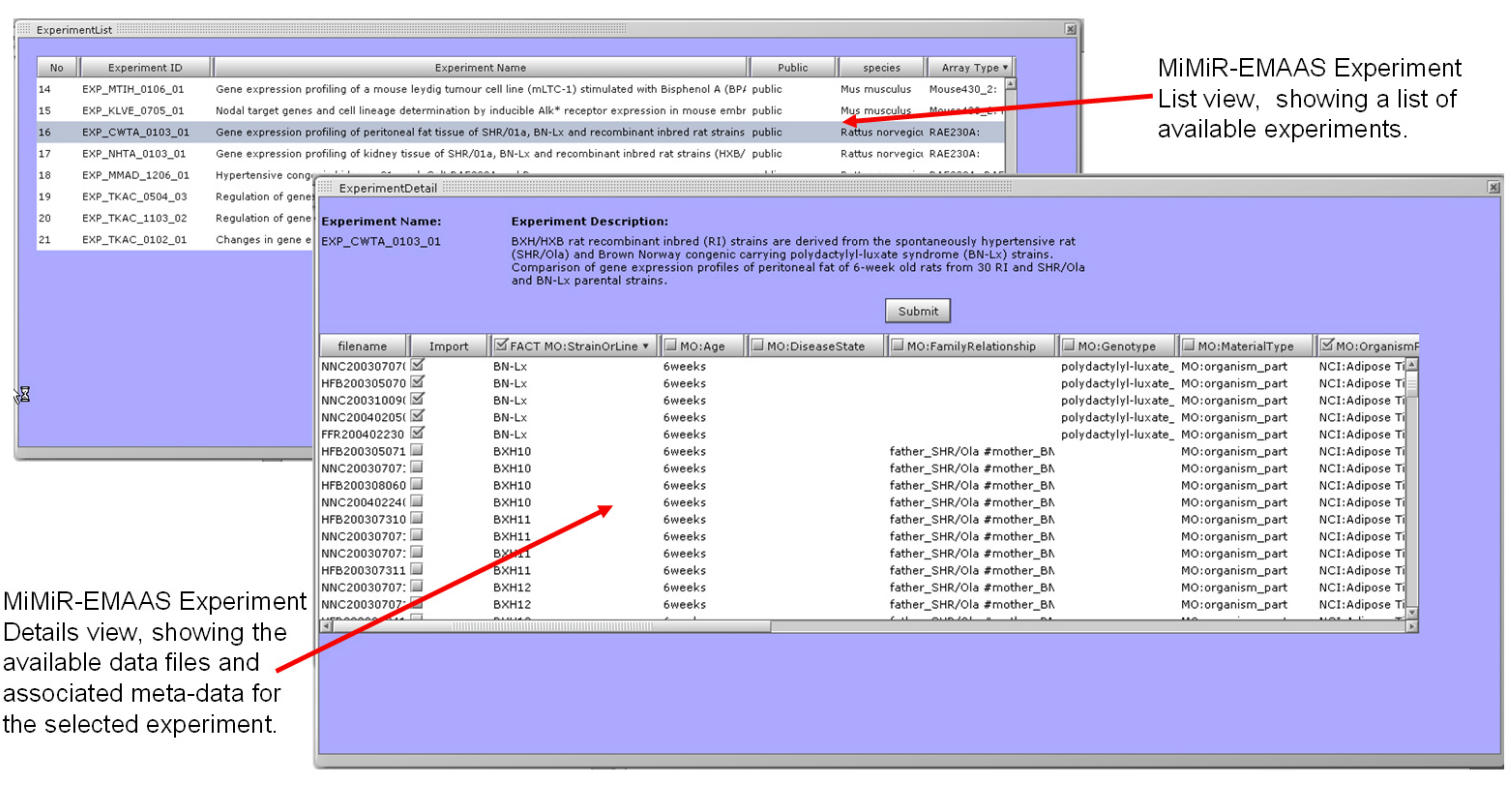
Screen shot of MiMiR – EMAAS interface, showing a guest user viewing and selecting data and meta-data from one of the publically available MiMiR experiments.

MiMiR is a MIAME-compliant microarray data warehouse supporting over 200 research group at the MRC Clinical Science Centre/Imperial College and 2 international consortia [25]. MiMiR provides a secure environment for collection, capture, comprehensive and consistent experimental annotation and dissemination of data [26]. It is fully integrated with EMAAS through the EMAAS-MiMiR web interface, see Figure 3 for screenshots.

The EMAAS web interface exercises the MiMiR middleware programming interface – a collection of java classes and methods developed to allow secure access to information in MiMiR from 3^rd ^party applications, such as EMAAS. A user may query and export experimental data and metadata that they have access rights to, including data which has been made publically available from MiMiR. A user may select all, or a subset, of Affymetrix .cel files from a MiMiR experiment. Cel files from different experiments for the same array type can be imported into single EMAAS analysis. Once a selection has been made the .cel files and associated user selected meta-data is then imported via the MiMiR middleware layer into the EMAAS environment for processing and analysis.

Users can also import data from the Gene Expression Omnibus (GEO)[27] without leaving the EMAAS interface. A user selects the Affymetrix platform type from a drop down menu and can enter an optional keyword to limit the search. EMAAS communicates in real time with the GEO server using the GEO web services and a list of experiments are returned and displayed to the user. The experiments are filtered to display only those that have Affymetrix cel files associated with them and contain the user's keyword in the title or experiment description.

Data can also be imported directly from the online public Affymetrix data repository CELSIUS[28]. As well as collating the raw data .cel files and associated meta-data from ArrayExpress[29]and GEO, CELSIUS also regularly reaps data from smaller public data repositories such as UCLA Los Angeles DNA Microarray Core Facility, MIT/Broad Institute and University of Pennsylvania Microarray Core Facility, with very little overlap of datasets between them. EMAAS communicates in real time with the CELSIUS data repository via the CELSIUS web services.

#### Experimental Design

An EMAAS Project is a collection of the same array types, allowing arrays to be collated into the same project from several sources. An experimental design describes which arrays belong to which biological factors. One or more experimental designs can be created within the same project. The experimental design view uses the interactive features of OpenLaszlo to drag and drop arrays to set up the biological factors that are to be compared. This dynamically builds up an XML dataset describing the experimental design.

When the experimental design has been set up and saved in the EMAAS interface, the XML schema describing the design is sent to the server and propagated through the data analysis workflow to suggest suitable pre-processing and analysis methods. The experimental design XML is transcribed into an R phenoData file on the server which is then used to read the data into R and to create the expressionSet data object. This expressionSet is serialized to the database for persistence and also saved as a global variable in the R-session for speed of access during the current analysis session.

#### Quality Assessment Analysis

A further advantage of using R-Bioconductor for microarray data analysis is that it has a large number of useful quality assessment (QA) plots available such as boxplots, PCA plots, simpleAffy[30] as well as others also found in the RReportGenerator[31] and arrayQualityMetrics packages[32]. However, these plots can take some time to generate – from a few seconds up to several minutes each – which can tie-up the analysis resources.

To overcome this problem grid compute clusters are used to generate the plots in parallel using the GridSAM and Grid Engine tools. GridSAM is used to pass data and launch the QA plot jobs on a Grid Engine-managed cluster, with each of the nodes having a locally installed instance of the R-Bioconductor tools. The data, experiment description, phenoData text file and a QA R script are passed to a compute node and R is invoked. The resulting QA plot is returned to the EMAAS database and the image displayed in the user interface. The small overhead in time required to copy data is negated by the saving in time it takes to produce all the plots, and the freeing up of resources for other analysis tasks. This system is scalable to allow other computationally intensive analysis algorithms to be integrated in a modular manner.

A further issue with the R plots that are generated is the lack of user interaction, the plots are often static images, and it is sometimes difficult to determine which arrays or data points are the outliers. To overcome this, the data used to generate the charts is passed back to the client interface as xml, which is then used to generate interactive Flash charts.

#### Data Pre-processing & Normalisation

##### Affymetrix 3' Gene Array

There are several pre-processing steps available in R, including MAS5[33] and RMA[34]. After the user has selected a method from the interface, the experimental design expressionSet object is obtained from the PLR session global variable, or unserialized from the database. This object is pre-processed using the selected algorithm and the resulting R dataframe is assigned to a PLR global variable, as well as being serialized to the database.

##### Exon Array

The EMAAS GridSam-GridEngine architecture has also been used to facilitate Affymetrix Exon array analysis. Affymetrix Power Tools (APTs) installed on the grid cluster nodes are used to process the raw data files. From the EMAAS web interface, the user selects the pre-processing algorithm of choice, e.g. RMA, PLIER[35], MAS5, and the summarisation level – either gene or exon, which is used to construct the APT command. The raw data .cel files are automatically transferred to the grid cluster and processed using APTs. The resulting summarised data file is returned to the EMAAS server, imported into R, the expressionSet object is generated and serialized to the database.

#### Differential Expression Analysis

The pre-processed expressionSet object can then be analysed to detect differentially expressed genes or exons using the Bioconductor Limma[36] or Exonmap[37] analysis packages through the EMAAS interface. The infrastructure is in place to allow further analysis algorithms developed within R, or using another technology to be added as required.

Results are passed back to the EMAAS interface as xml and displayed in an interactive table, allowing users to sort data, highlight data and select which columns of information and data are displayed in the table. Double clicking on a gene row generates an interactive expression profile plot and selecting on the plot pulls up the relevant GeneCard [38] for the selected gene. GeneCards is an online accessible database which provides a vast array of information on all known and predicted human genes.

Users can also copy and paste selected rows of interest into 3^rd ^party applications, such as Microsoft Excel, for further analysis.

#### Functional Annotation Analysis

Gene annotations are retrieved automatically from the R annotation library packages if available. The R-Biomart package [39] can also be used to retrieve annotation information for genes of interest. Exon annotation is retrieved from an EMAAS database table built using the Affymetrix annotation files [40]. This requires periodic rebuilding as and when Affymetrix update their annotation files.

Gene lists generated during analysis can be passed directly from the EMAAS interface to DAVID (The Database for Annotation, Visualization and Integrated Discovery), a web site for functional enrichment analysis, which enables the discovery of biological groups of potential interest associated with a particular gene list. Submission to DAVID is accomplished through a modified version of the public DAVID programming API. The API allows users to submit a list of genes with all relevant parameters and directly analyze the list in any of DAVID's analysis modules. This differs from the typical approach of using DAVID by automatically handling the list submission, list property selection, and workflow normally required to begin using a given analysis module. In addition, the modified version of the API does not restrict list size, number of submissions, and duration between submissions as does the public version of the API.

#### Download data and Export of Results

The R workspace object for any of the experimental designs can be downloaded from the EMAAS interface. This enables users to continue analysis in their local installation of R if so desired. User-selected data from the results table can also be copied to the clipboard for use in 3^rd ^party applications

## Results

### See table 1 for a full list of resources integrated into EMAAS

The current local version of the EMAAS web server is deployed on a server configured with two dual-core AMD Opteron processors and 8 Gb RAM running Apache Tomcat. The database server has two dual-core AMD Opteron processors and 32 Gb RAM. The database is stored on a local RAID 10 array providing 1.8 Tb of storage. Both the database and web server are running the Red Hat Enterprise 5.2 operating system.

The grid cluster currently used as execution hosts, hosted by the London e-Science Centre, has 248 compute nodes, each with a dual core 2 GHz or 2.8 GHz P4 processor, 2 GB RAM and a Fast Ethernet connection.

EMAAS has been successfully tested with 32 demonstration users concurrently logged into the system, and simultaneously downloading 100 Affymetrix Rat Genome 230 2.0 arrays from the MiMiR data warehouse, setting up experimental designs, RMA pre-processing the data, performing a t-test with multiple testing p-value corrections to determine differentially expressed genes, retrieving gene annotation and passing selected interesting gene lists to DAVID.

The database server has been successfully tested with 64 concurrent virtual users making simultaneous requests to RMA process 132 Mouse 430 2.0 Affymetrix arrays. RMA processing of the data is currently the most computationally intensive step of the data analysis workflow.

## Discussion

EMAAS has been successfully implemented and can be used by researchers to perform microarray data analysis at the gene level or exon level.

Although we are aware of the large numbers of microarray tools currently available, including the well established and utilised BASE[41], SMD[42] and MeV[43], we believe that the following combination of features of EMAAS gives it a unique place in the microarray data analysis field:

◦ Web based rich internet application – no installation requirements on the client side

◦ User friendly intuitive interface – drag and drop and interactive visual components facilitate the analysis pipeline

◦ All integrated resources are accessible from a single user interface, users do not have to learn how to use multiple interfaces

◦ Seamless integral access to grid compute resources – enabling improved performance and also facilities the scaling up of the system as more users come online

◦ User friendly intuitive interface – all integrated resources are accessible from a single user interface, users do not have to learn how to use multiple interfaces

◦ Integrated with the MiMiR microarray data warehouse for access to raw data and richly annotated meta-data

◦ Data and analysis results stored on centrally maintained server – no risk of loss of data on users local computer

◦ Integrated access to public data repositories (GEO and CELSIUS) allowing data in the public domain to be searched and imported for analysis with data sets previously imported into EMAAS

◦ Analysis tracking – each step of the workflow is captured, stored and can be revisited

◦ Data and analysis results can be shared between users

The EMAAS interface has been created in the RIA technology OpenLaszlo, to create a user friendly, interactive and intuitive web interface.

EMAAS has integrated R into its system. R, in combination with Bioconductor, is considered to be one of the most powerful, flexible and most widely used microarray data analysis tools, yet at the same time can be difficult for the beginner to use. It is driven from the command line and has its own object oriented programming language which is not intuitive, particularly for those who are not familiar with programming concepts or statistical methodologies. EMAAS allows users access to the wealth of microarray functionality available in R.

However, EMAAS analysis functionality is not just limited to R. The EMAAS architecture has been designed to allow other tools to be added where appropriate in a modular fashion, either located locally, such as the APTs, or located at remote facilities such as DAVID and CELSIUS.

Learning the practicalities of undertaking meaningful microarray data analysis is a non-trivial subject area. EMAAS has been designed to facilitate distance support interactions between bioinformatics support services and users in their microarray analysis. Support staff can communicate in real-time with users using the web conferencing tool EVO. The administration interface allows support staff to access the user's data where required, whilst maintaining secure password-protected user and group-access designations.

### Future Directions

Although current analysis functionality is somewhat limited to the building blocks of standard Affymetrix data analysis workflows, the framework already in place is flexible and scalable so that new data analysis algorithms and methods can be integrated with relative ease. Additional analysis methods will be added according to user requirements in the beta testing phase. The functionality to analyse imported data from different array platforms such as Agilent, Illumina and custom 2-colour arrays is already available in R, as is the functionality for the analysis of other array methodologies such as SNP chip and ChIP-chip arrays.

EMAAS can currently be extended to use these technologies. In the present version new analysis methods are added via the server side technologies. However development work is currently being carried out to allow users to add new methodologies and R scripts directly via the EMAAS web interface.

Work is already underway to allow users to create and re-use data analysis workflows, to add extra functionality for functional enrichment analysis and to add methodologies for co-expression analysis.

## Conclusion

EMAAS is a user-friendly, flexible and scalable microarray data analysis platform, which integrates several commonly used data analysis tools including R-Bioconductor, Affymetrix Power Tools and DAVID, making them accessible through a single web interface. EMAAS has been designed with user support in mind to aid users through their data analysis.

## Availability and requirements

**• Project name: **EMAAS

**• Project home page: **

**• Operating system(s): **interface: cross-platform; Server side: Linux

**• Programming language: **Java, OpenLaszlo, R

**• Other requirements: **R, Postgres, PLR, GridSAM, Apache-Tomcat, OpenLaszlo

**• License: **A tar archive containing binaries for server-side remote installation of the EMAAS system on Linux operating systems is available for download from .

The user interface is publically available at 

MiMiR can be accessed via MiMiR Online from the Microarray Centre-MiMiR User Centre web pages .

## Abbreviations

API: Application Programming Interface; APTs: Affymetrix Power Tools; ChIP-chip: Chromatin immunoprecipitation on a microarray chip; DAVID: The Database for Annotation, Visualization and Integrated Discovery; EVO: Enabling Virtual Organizations; MIAME: Minimum Information About a Microarray Experiment; MiMiR: Microarray data Mining Resource; PDF: Portable Document Format; PLR: postgres Procedural Language for R; QA: Quality Assurance; RIA: Rich Internet Application; SNP: Single Nucleotide Polymorphism; XML: eXtensible Markup Language.

## Authors' contributions

GB designed and developed the EMAAS user interface, designed and developed the EMAAS Postgres DB, Wrote PLR functions and integrated APTs and video tutorials. MK wrote the installation scripts, QA pdf report generator and was involved with the integration of the distributed computing tools. NC developed the EMAAS query interface for MiMiR, GEO and CELSIUS and integrated the administrative interface. JMS Developed the parallel computing code, including running QA jobs on the GridEngine-Managed Cluster vie GridSAM, and developed the Administrative interface.

JA set up and maintained the project development and deployment servers and also set up and maintains the project website. AS set up the project infrastructure tools including SVN, ant and the project wiki. BT Initiated the EMAAS-MiMiR interface. YH Initiated the GridSAM Parallel computing code.

SAB first conceived the idea of the system together with MJS, led the project, and together with CT, MS, TA, LG & JD provided insights on software development and testing and critically reviewed the manuscript.
